# Are People From Black Communities Proportionately Represented in UK and US Studies Examining Views on Screening and Diagnostic Genetic Testing in Pregnancy? A Scoping Review

**DOI:** 10.1111/1471-0528.18195

**Published:** 2025-04-29

**Authors:** Michelle Peter, Rashida Baptiste, Celine Lewis, Lyn S. Chitty, Melissa Hill

**Affiliations:** ^1^ North Thames Genomic Laboratory Hub, Great Ormond Street Hospital for Children NHS Foundation Trust London UK; ^2^ Genetics and Genomic Medicine UCL Great Ormond Street Institute of Child Health London UK; ^3^ Population, Policy and Practice Department UCL Great Ormond Street Institute of Child Health London UK

**Keywords:** African American, attitudes, black people, decision‐making, health equity, inequities, prenatal diagnosis, prenatal testing

## Abstract

**Background:**

Several studies have explored parent and public perspectives on screening and diagnostic genetic testing during pregnancy (prenatal testing). Little is known about how much people from Black communities have contributed to this research.

**Objective:**

To examine whether Black people's views on prenatal testing are proportionately represented in UK and US studies.

**Search Strategy:**

Searches were conducted in Medline (Ovid), PsycINFO (Ovid), CINAHL Complete (EBSCOhost), and Emcare (Ovid).

**Selection Criteria:**

Primary experimental UK and US studies examining parental and public perspectives on prenatal testing, published between 2014 and 2024.

**Data Collection and Analysis:**

After duplicate removal, titles and abstracts were independently screened by two reviewers. Full texts were then obtained, and data were extracted for analysis.

**Main Results:**

Seventy‐six studies were included. 83% (*n* = 63) included Black participants; only 39% (*n* = 30) reported a sample meeting the respective national Black population. More studies in which Black participants met the population were from the UK (UK: 69% vs. US: 42%), though this difference did not reach significance (OR = 1.53; 95% CI: 0.52, 4.48; *p* = 0.431). Black participants were better represented in studies exploring views on prenatal testing for sickle cell than those on non‐invasive prenatal testing and genomic technologies.

**Conclusions:**

Whilst important for our understanding, efforts to include Black participants in studies examining views on prenatal testing should not be limited to those where the condition primarily impacts this population. Improved representation of Black people across a wider range of studies is essential for supporting health equity and minimising health disparities.

## 1 Introduction

Prenatal tests are offered in pregnancy to identify the risk and presence of fetal anomalies. Though these tests play a crucial role in pregnancy management, neonatal care, and long‐term prognoses [1, 2], they can have significant emotional and psychological impacts for expectant parents [3, 4] and present considerable challenges for parental decision‐making [5].

High quality counselling for parents during this time is essential to support them to make decisions that are right for them. This requires that healthcare professionals (HCPs) recognise that the support needs for different populations may vary. For instance, parents from Black communities have been noted as having higher rates of distress in response to a fetal anomaly compared to parents from other ethnic backgrounds [6], underscoring the value of targeted support. HCPs should also be equipped to provide counselling that aligns with parents' core values [7], particularly since decision‐making for obstetric interventions in some communities may be guided by religious and cultural beliefs [8, 9]. Studies examining views and experiences of screening and diagnostic genetic testing during pregnancy (hereon in referred to as ‘prenatal testing’) can provide important insights into what this support should look like. It is critical that these studies capture the perspectives of people from diverse ethnic and cultural backgrounds as understanding the needs of different communities is essential for guiding the development of equitable prenatal testing services.

A key issue, however, is that Black people are underrepresented across many health and social research contexts [10]. Racial health inequalities, driven by structural racism [11, 12, 13] and the intersection of race and ethnicity with other social determinants of health [14, 15] also mean that Black women in the UK and US face disproportionately higher risks of maternal mortality and morbidity compared to women from other racial and ethnic groups [16, 17]. In effect, people from Black communities face some of the worst health outcomes, yet are the least represented in research studies.

Awareness of these disparities is growing [18]. However, no research, to our knowledge, has examined the extent to which Black people are represented in studies examining views on the screening and diagnostic genetic tests offered in pregnancy. Identifying where gaps in the literature exist on this topic is essential for guiding future research. Thus, the current scoping review aimed to answer two key questions: (1) What proportion of UK and US studies include the views of Black people? (2) Of these studies, what proportion of participants do Black people make up?

## 2 Methods

A scoping review was deemed the most appropriate method for answering the research question since this approach is ideal for mapping the literature and identifying key gaps in research. The review was guided by the Joanna Briggs Institute for Scoping Reviews methodology [19] and the Reporting Checklist for Scoping Reviews (PRISMA‐ScR) [20]. The study protocol has been registered in Open Science Framework (https://doi.org/10.17605/OSF.IO/7ZEGD).

### 2.1 Eligibility Criteria

**FIGURE 1:**
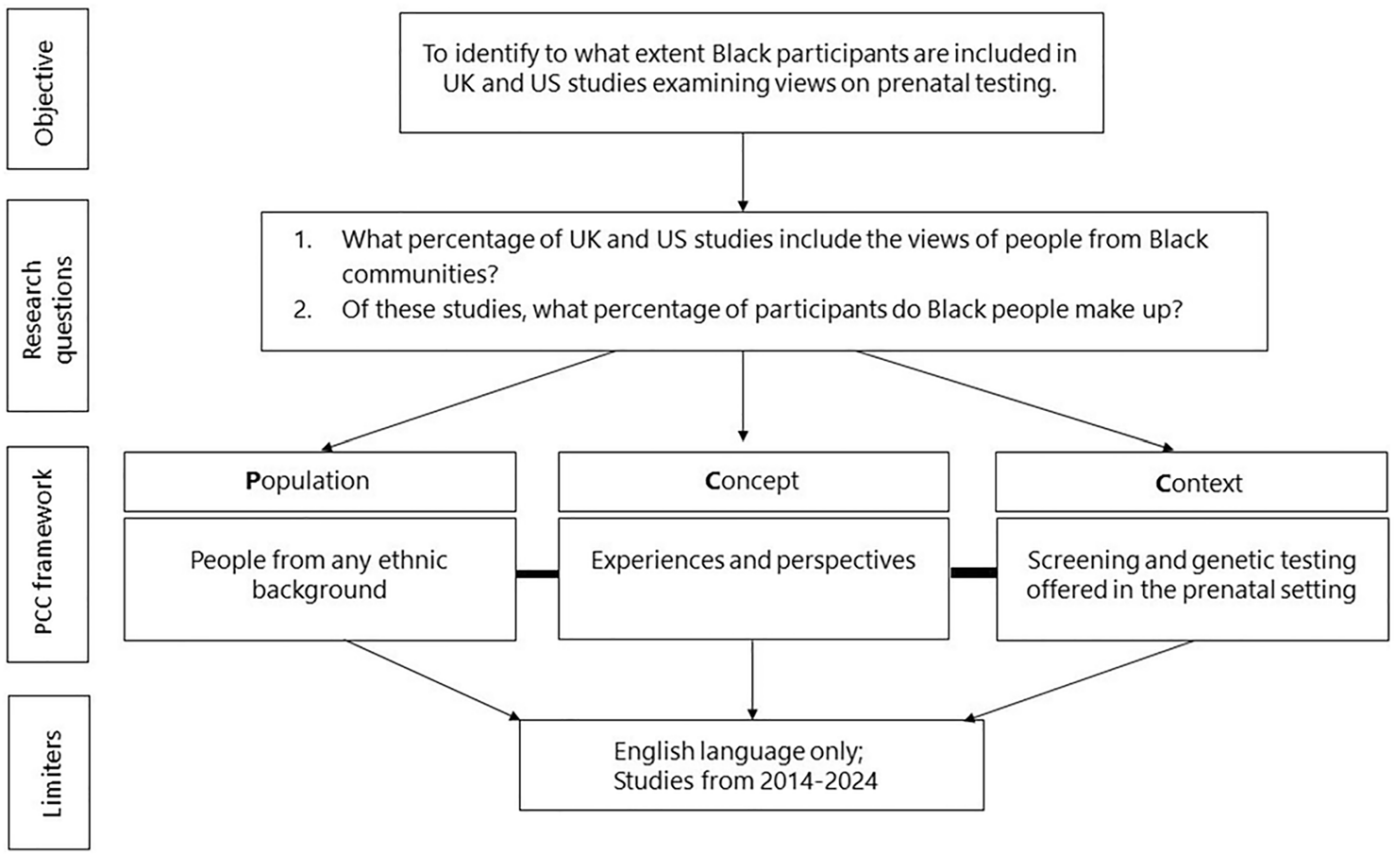
PCC framework used to define the search strategy.

The Population Concept Context (PCC) [21] framework (Figure 1) was used to develop the research question and the inclusion and exclusion criteria (Table 1). Any primary experimental UK or US study examining parental experiences and perspectives on screening or diagnostic genetic tests offered in pregnancy, published in English between 2014 and 2024, was considered eligible. We focused exclusively on UK and US studies because, in both countries, Black women face the greatest risk of adverse maternal outcomes [16, 17]. Any screening or diagnostic genetic test offered in pregnancy, including ultrasound, was considered prenatal testing. We considered Black people as anyone reported in the literature as Black, Black British, Black African, Black Caribbean, African American, Black Hispanic, or coming from other Black or mixed Black heritage. However, it is duly acknowledged that Black people are not a monolithic group and that there is an array of ethnicities to which people may choose to identify. Only studies where the ethnicity of participants was reported, allowing disaggregation from other ethnic groups, were included in the final review.

**TABLE 1:** Inclusion and exclusion criteria.

Included studies	Excluded studies
Studies published between 2014 and 2024	Studies outside of the UK or US
Studies that assess parent or public views, attitudes, knowledge, understanding, decision‐making, experiences, perceptions, and perspectives on prenatal testing	Studies reporting experiences and perspectives on screening or genetic testing outside of the prenatal setting
Studies that use quantitative, qualitative, or mixed methods design	Studies only reporting perspectives of healthcare professionals
Studies published in English	Studies where the ethnicity of participants has not been included to allow disaggregation from other ethnic groups within the study
	Studies where the focus is on people from an ethnic group that is not a Black ethnic group (e.g., a study focussed on Asian women)
	Studies exclusively focused on test development, diagnostic yield and clinical utility
	Case reports, opinion pieces, review articles, and systematic/scoping reviews
	Studies with a molecular focus

### 2.2 Search Strategy and Study Selection

A comprehensive search strategy was developed in consultation with a research librarian. An initial search in Medline (Ovid) was conducted to identify relevant keywords and subject headings. These terms were used to develop a full search strategy where they were combined using Boolean operators to produce a search string (Appendix S1). This search strategy was adapted for the following three databases: (1) PsychINFO (Ovid), (2) CINAHL Complete (EBSCOhost), and (3) Emcare (Ovid). A manual search of reference lists from all studies included at the full text screening stage was conducted.

### 2.3 Data Extraction

**FIGURE 2:**
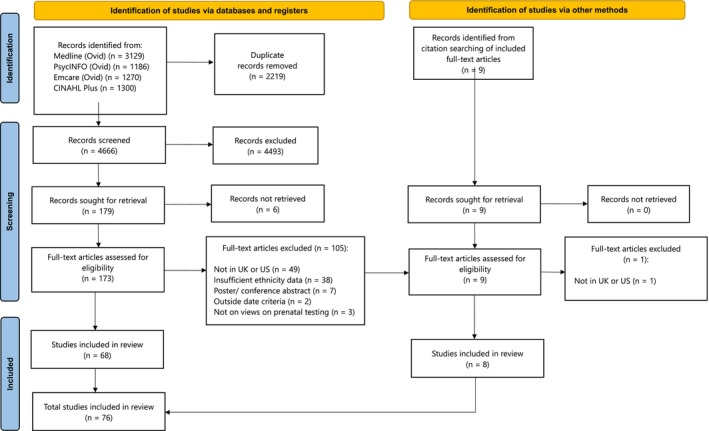
PRISMA flow diagram of study selection process.

All identified citations were collated and imported into Rayyan review management software (http://rayyan.qcri.org) where duplicates were removed. Two reviewers (MP, RB) independently screened titles and abstracts for 20 sources to test that the inclusion and exclusion criteria were being applied consistently. Once satisfied, the remaining titles and abstracts were screened independently by both researchers. Full texts were obtained for included studies, and discrepancies were resolved through discussion until consensus was reached. The study selection process is outlined in a PRISMA flow diagram (Figure 2). Data from included articles were entered into a data extraction form by two reviewers (MP, RB). The form was piloted on five sources to ensure its suitability. Any disagreements were resolved through discussion. The following study characteristics were collected: bibliographic information, country of publication, study aim, population, prenatal test type, genetic condition, methodology, total sample size, sample size of Black participants, and ethnic/racial groups included in the study.

### 2.4 Data Analysis and Presentation

To summarise the data, descriptive statistics including frequencies and percentages were reported. Odds ratios were calculated to quantify the strength of the association between relevant variables. The percentage of Black participants in studies was compared against the national Black population of women giving birth in the UK [22] (2022) and the US [23] (2020–22).

## 3 Results

### 3.1 Screening Process

Our search identified 6885 articles across the four databases. After 2219 duplicates were removed, 4666 articles were put forward for title and abstract screening. Of these, 4493 were excluded. Six articles could not be retrieved and so 173 were included for full text screening. At this stage, 105 articles were excluded because the study: (a) was not based in the UK or US (*n* = 49); (b) was presented as a conference abstract or poster with limited information (*n* = 7); (c) was outside the date threshold (*n* = 2); (d) did not focus on views on prenatal testing (*n* = 3); (e) did not include sufficient information to determine the ethnic makeup of the study sample (*n* = 38), such as failure to report participant ethnicity (*n* = 20) and reporting only a White ethnic group as a normative category (*n* = 13). Thus, 68 articles were identified for inclusion in the review. A manual search of the references of these 68 articles by the first author (MP) identified a further nine articles which were independently screened and approved by a second reviewer (RB). One article was excluded because it was not based in the UK or US. As such, a total of 76 studies was included in the final review—all of which included participant ethnicity, allowing us to assess representation of Black participants.

### 3.2 Study Characteristics

Across the 76 studies, we noted an even proportion of qualitative and quantitative studies. Around three quarters of studies (74%, *n* = 56) originated from the US, and 46% (*n* = 36) were published between 2014 and 2016. Whilst 53% (*n* = 42) of articles did not focus on a specific condition, 22% (*n* = 17) explored views and experiences of prenatal testing for Down syndrome and other aneuploidies. The most frequent testing method examined was non‐invasive prenatal testing (NIPT) which was the focus of 27 studies. A summary of study characteristics can be seen in Table 2. Detailed information about each included study can be found in (Table S1).

**TABLE 2:** Study characteristics.

	*N* (%)		*N* (%)
Country where study was conducted		Total sample size	
US	56 (74)	< 20	11 (14)
UK	17 (22)	21–40	18 (24)
UK + others^a^	2 (3)	41–60	8 (11)
US + others^b^	1 (1)	61–100	8 (11)
		101–200	9 (12)
Year of publication		201–1000	16 (21)
2014	11 (14)	> 1000	4 (5)
2015	14 (18)		
2016	11 (14)	Type of prenatal test	
2017	5 (7)	Amniocentesis	2 (3)
2018	3 (4)	NIPT	27 (36)
2019	6 (8)	NIPD	3 (4)
2020	8 (11)	Exome/genome sequencing	10 (13)
2021	4 (5)	Microarray	4 (5)
2022	6 (8)	Diagnostic testing generally	4 (5)
2023	4 (5)	Prenatal screening	11 (14)
2024	4 (5)	Prenatal testing generally	13 (17)
		Not stated	2 (3)
Methodology used			
Qualitative	35 (46)	Type of condition^c^	
Quantitative	33 (43)	Downs syndrome/other aneuploidies	17 (22)
Mixed methods	8 (11)	Sickle cell/other blood conditions	9 (11)
		Auto recessive conditions	3 (4)
Method of data collection		Single gene disorder	2 (3)
Interviews	31 (41)	CHD	2 (3)
Surveys/questionnaires	34 (45)	Developmental condition	1 (1)
Focus groups	3 (4)	Structural anomaly	1 (1)
Multiple methods	8 (11)	Differences of sex development	2 (3)
		Not stated	42 (53)

^a^ Only UK demographic information reported, so categorised as UK for analyses.

^b^ Only US demographic information reported, so categorised as US for analyses.

^c^ Total number of conditions exceeds total number of papers because some papers included more than one condition.

### 3.3 Representation of Black Participants Across UK and US Studies

Across all 76 studies, where participant ethnicity was reported, 83% (*n* = 63) included Black participants. However, the percentage of Black participants included in UK studies (68%, *n* = 13; median = 4, IQ1 = 0, IQ3 = 8.5) was lower than that in US studies (88%, *n* = 50; median = 8, IQ1 = 3, IQ3 = 18) (Table S2). This was corroborated by the finding that, whilst non‐significant, UK studies had 70% lower odds of including Black participants compared to US studies (OR = 0.30; 95% CI: 0.09, 1.13; *p* = 0.080). Despite this, more UK than US studies included a sample size of Black participants that met the national Black birthing population (based on national data relating to the rate of women giving birth in the UK [22] and the US [23]). Black participants were adequately represented in 47% (*n* = 9) of UK studies compared to 35% (*n* = 20) of US studies. Although not reaching significance, this was in line with the finding that UK studies had 61% higher odds of representing the national Black population compared to US studies (OR = 1.66; 95% CI: 0.56, 4.84; *p* = 0.357).

Notably, whether a study included a sample that met the national Black population varied with the condition in question (where stated). For instance, six out of seven studies (86%) exploring attitudes towards prenatal testing for sickle cell (SC) included a sample that met the national Black population [24, 25, 26, 27, 28, 29]. In comparison, only 35% examining attitudes towards testing for Down syndrome and other aneuploidies did so [30, 31, 32, 33, 34, 35], whilst two studies examining views on testing for monogenic disorders [36, 37] or one examining structural anomalies [38] included no Black participants at all.

Twenty‐seven studies focused on NIPT as a screening method: seven from the UK [32, 33, 34, 35, 39, 40, 41] and 20 from the US [24, 31, 42, 43, 44, 45, 46, 47, 48, 49, 50, 51, 52, 53, 54, 55, 56, 57, 58, 59]. Of these, Black participants were underrepresented in 16 studies (59%): three from the UK [39, 40, 41] and 13 from the US [42, 43, 44, 45, 47, 51, 52, 53, 55, 56, 57, 58, 59]. Views on prenatal exome and genome sequencing were examined in 10 studies [36, 60, 61, 62, 63, 64, 65, 66, 67, 68], but only two of these included a sample representative of the Black population: one from the UK [68] and one from the US [63]. Three studies examined views on non‐invasive prenatal diagnosis (NIPD). Of these, two did not include a sample that met the national Black population [37, 69]. The study that did included SC as one of the conditions of focus [26].

In 10 studies, there was specific mention of trying to recruit people from a range of backgrounds. In five studies it was stated that the sampling strategy sought maximum diversity in terms of ethnicity [25, 41, 49, 70, 71]; one study specifically ascertained the views of African American women [24]; one focussed on the Somali community [72]; one aimed to recruit from five ethnic groups [73]; one stated that ethnicity was tracked in order to “appraise equity in enrolment and uptake” [65]; and one contracted a web‐based survey provider to recruit a sample representative of the primary demographic features of the US [52]. Despite these efforts, Black participants remain underrepresented in three of these studies [41, 52, 65].

## 4 Discussion

### 4.1 Main Findings

This review aimed to identify whether Black people are proportionately represented in UK and US studies examining experiences and perspectives on prenatal testing. We found that many studies have included Black participants and that a greater proportion of these studies are from the US. However, whilst Black people were found to have participated in most studies, their inclusion was often not proportionate to the respective UK and US Black birthing populations. Notably, this pattern varied depending on a study's focus: Black participants were overrepresented in studies exploring views on prenatal diagnosis for sickle cell (SC) but underrepresented in studies gathering insights on non‐invasive prenatal testing (NIPT), non‐invasive prenatal diagnosis, and genomic technologies.

### 4.2 Strengths and Limitations

A strength of this work is that we employed an exhaustive search strategy and a rigorous approach to study selection. A limitation is that we were unable to include studies that would have fit our inclusion criteria but that did not provide sufficient racial and ethnic participant information. We also cannot be sure how racial and ethnic data were determined across studies; some may have been self‐reported, and some ascribed by the researchers, which could affect accuracy. This review is also limited to UK and US studies. Finally, it was not within the scope of this review to address potential reasons for the underrepresentation of Black participants. However, we recognise that, for meaningful change in research practice, disparities in research participation must be contextualised against an understanding of how historic and current systems have shaped present‐day inequities.

### 4.3 Interpretation

This scoping review revealed that Black participants are underrepresented in the majority of UK and US studies examining experiences and perspectives on prenatal testing. This finding was prevalent in studies focused on NIPT, which made up 36% of studies in this review. NIPT may be viewed favourably because of its high sensitivity and specificity [74]. However, its limitations include the possibility of false‐positive and false‐negative results, and the need for diagnostic confirmation via invasive methods following a positive result [75]. Researchers have, therefore, been keen to gather public insights into NIPT and the way it is offered. Nonetheless, our review identified that many studies lack adequate input from people from Black communities, and so our understanding of the factors that shape their decision‐making and support requirements following a positive result from NIPT is limited. Notably, NIPT has been available commercially in the UK and the US since 2011 [76], and in June 2021, it was implemented into routine care via England's National Health Service (NHS). Thus, the potential for global adoption of NIPT into clinical settings has long been anticipated. A key question, therefore, is why much of the research examining its acceptability has failed to proportionately capture the views of people for whom maternal and pregnancy outcomes are amongst the worst [13, 17, 77]?

We observed a similar pattern in studies on attitudes towards prenatal exome sequencing (pES) and genome sequencing; only two of ten studies included a representative sample of Black participants [63, 68]. In England, pES has been part of routine care since October 2020, delivered through the NHS Genomic Medicine Service (GMS) [78]. The GMS was designed to ensure equitable access to genetic tests and research participation. However, this review highlights minimal contributions from Black participants in research on attitudes towards pES, raising uncertainties around whether current counselling approaches for pES meet the needs of Black communities. Without addressing these gaps, healthcare professionals may offer care that is equal but not equitable, further exacerbating existing disparities in the UK and US.

In contrast, we observed a greater proportion of Black participants in six of the seven studies on SC [24, 25, 26, 27, 28, 29], with Black people making up around 73% of the sample. Whilst expected, given SC's prevalence amongst those of African and Caribbean descent, with increasing migration and diversity of populations, the condition can affect people from any ancestral group. Moreover, genetic conditions other than SC are relevant in pregnancy to Black people. Relatedly, the only study assessing views on NIPD where Black participants were adequately represented included SC as one of the conditions of focus [26]. Only hearing from Black participants when the study focuses on SC reinforces views of this condition as a “Black disease” [79] and perpetuates a cycle of exclusion and inequality in research.

The underrepresentation identified in this review is not unique to prenatal research. A recent NHS Race & Health Observatory report highlighted that genetic studies more broadly lack data from global majority communities [80], leading to biased genetic databases and a distorted understanding of genetic diversity [81]. Additionally, our knowledge of the personal and clinical utility of genomics lacks diverse participant experiences [82], further exacerbating health inequalities.

Prenatal diagnosis is evolving, and new techniques are increasingly integrated into clinical settings. Capturing the views of Black communities is crucial as all parents who encounter prenatal testing deserve a role in shaping how tests are offered, how results are communicated, and how emerging technologies are used. An additional important consideration in the US is that access to prenatal testing is governed by health insurance coverage which is amongst the lowest for Black people [83]. We observed that inclusive approaches to recruitment have already been adopted; attempts to widen participation were mentioned in a small number of studies in this review [24, 25, 41, 49, 52, 65, 70, 71, 72, 73]. However, work is still needed. Thirteen studies were excluded from our review for including ‘White’ as the default ethnic category. This practice obscures our understanding of diversity and inclusion in research whilst perpetuating the narrative of White populations as the norm against which everyone is measured. Evidently, researchers have a pivotal role to play in ensuring Black people are adequately represented in studies.

## 5 Conclusion

Although many UK and US studies on parent and public views of prenatal testing have included Black participants, their insights are not proportionately captured unless the focus is on sickle cell. There is limited understanding of Black people's perspectives on prenatal testing outside the context of sickle cell, despite Black women facing the highest risk of adverse maternal and pregnancy outcomes. Concerted efforts must be made to improve the representation of Black people across all studies on prenatal testing. This is crucial for promoting health equity, reducing disparities, and ensuring that Black people's insights are considered at what can be a pivotal stage in pregnancy.

## 6 Practical and Research Recommendations

Black perspectives on NIPT and NIPD remain underexplored, despite important ethical concerns [84, 85]. Future research must prioritise understanding Black communities' attitudes to ensure equitable care.

Expanding research participation requires change at multiple levels. At the individual level, we suggest researchers collaborate with trusted advocates [86] and establish advisory groups with meaningful representation of Black individuals to promote culturally sensitive study design and implementation. At the structural level, we suggest increasing the racial and ethnic diversity of researchers where possible. Not only can researchers from different backgrounds build trust through researcher‐participant concordance, but they may also prioritise studies that address their communities [87, 88]. This need has already been recognised, with some funding bodies creating targeted funding streams for Black researchers to address current inequities in academia [89].

Finally, we recommend transparent reporting of racial and ethnic data. This is essential for contextualising findings and monitoring diversity and inclusivity within research.

## Author Contributions

M.P. conceived the study. M.P., C.L., and M.H. designed the study. M.P. performed the systematic search. M.P. and R.B. conducted the screening process, data extraction, and data management. M.P. conducted the statistical analyses. M.P. wrote the first and final draft of the manuscript. M.P., C.L., M.H., and L.S.C. contributed to the interpretation of results. All authors contributed to manuscript revision.

## Ethics Statement

The authors have nothing to report.

## Conflicts of Interest

The authors declare no conflicts of interest.

## Supporting information


**Appendix S1.** Search strategy for the four databases, including planned limits.

**Table S1.** Study characteristics of all studies included in the final review.

**Table S2.** Percentage of Black participants included across studies.

## Data Availability

The data that supports the findings of this study are available in the Supporting Information of this article.
