# Supplementary material for: Are People From Black Communities Proportionately Represented in UK and US Studies Examining Views on Screening and Diagnostic Genetic Testing in Pregnancy? A Scoping Review
Source: BJOG. 2025 Apr 29;132(13):1956–65. doi: 10.1111/1471-0528.18195 (PMC12592762; doi:10.1111/1471-0528.18195)
Supplement: Supplementary file 1 — Appendix S1. Search strategy for the four databases, including planned limits. [file BJO-132-1956-s003.docx]

**Appendix S1.** Search strategy for the four databases, including planned limits.

1. **Medline (Ovid) with the filters: English language; from 2014-current:**

Keywords: (parent* or women* or couples or public or communit* or American* or Latin*) OR Subject heading terms: (Parents [px] OR “black or african American” OR “hispanic or latino" OR Pregnant Women)

AND

Keywords: (experiences OR attitude* OR awareness OR perspective* OR belief* OR decision* OR preference* OR view*) OR Subject heading terms: (Attitude OR attitude to health OR health knowledge, attitudes, practice OR patient acceptance of health care OR patient satisfaction OR Decision Making)

AND

Keywords: (Down* syndrome adj3 screen* OR trisom* adj3 screen* OR aneuploid* adj3 screen*) OR (NIPT or microarray or array CGH or fetal exome or foetal exome or prenatal exome) OR ((prenatal or fetal or foetal) adj3 genom*) OR ((fetal or foetal) adj4 screen*) OR (pregnan* adj4 sickle cell) OR Subject heading terms: (Pregnancy [di, px] OR Prenatal Care [mt, px] OR Pregnancy Trimester, First [px] OR Pregnancy Trimester, Second [px] OR Prenatal Diagnosis [px] OR Noninvasive Prenatal Testing OR Anemia, Sickle Cell [di, ge, pc, px] OR Down Syndrome [di, pc])

1. **PsychINFO (Ovid) with the filters: English language; from 2014-current:**

Keywords: (parent* or women* or couples or public or communit* or American* or Latin*) OR Subject heading terms: (blacks OR "latinos/latinas" OR parents OR exp expectant mothers)

AND

Keywords: (experiences OR attitude* OR awareness OR perspective* OR belief* OR decision* OR preference* OR view*) OR Subject heading terms: (attitudes OR exp health attitudes OR exp parental attitudes OR decision Making)

AND

Keywords: (prenatal screen* or prenatal test* or prenatal diagnos* or antenatal screen* or antenatal test* or antenatal diagnos*) OR (Down* syndrome adj3 screen* OR trisom* adj3 screen* OR aneuploid* adj3 screen*) OR (NIPT or microarray or array CGH or fetal exome or foetal exome or prenatal exome) OR ((prenatal or fetal or foetal) adj3 genom*) OR ((fetal or foetal) adj4 screen*) OR (pregnan* adj4 sickle cell) OR Subject heading terms: (prenatal care OR prenatal diagnosis OR exp genetic disorders OR sickle cell disease OR exp anemia OR down’s syndrome)

1. **CINAHL (EBSCO) with the filters: English language; from 2014-current:**

Keywords: (parent* or women* or couples or public or communit* or American* or Latin*) OR Subject heading terms: (Parents OR "Black Persons+" OR "Americans+") OR "Expectant Parents+")

AND

Keywords: (experiences OR attitude* OR awareness OR perspective* OR belief* OR decision* OR preference* OR view*) OR Subject heading terms: ("Attitude to Health" OR "Patient Attitudes" OR "Decision Making, Patient+")

AND

Keywords: (prenatal screen* or prenatal test* or prenatal diagnos* or antenatal screen* or antenatal test* or antenatal diagnos*) OR (Down* syndrome N3 screen* OR trisom* N3 screen* OR aneuploid* N3 screen*) OR (NIPT or microarray or array CGH or fetal exome or foetal exome or prenatal exome) OR ((prenatal or fetal or foetal) N3 genom*) OR ((fetal or foetal) N4 screen*) OR (pregnan* N4 sickle cell) OR Subject heading terms: ("Prenatal Care/PF" OR "Prenatal Diagnosis/PF" OR "Noninvasive Prenatal Testing" OR "Anemia, SickleCell/PC/PF/DI" OR "DownSyndrome/DI/PC/PF")

1. **Emcare (Ovid) with the filters: English language; from 2014-current:**

Keywords: (parent* or women* or couples or public or communit* or American* or Latin*) OR Subject heading terms: (exp expectant parent/ OR exp expectant mother/ OR parent/ OR black person/ OR exp african american/ OR hispanic/)

AND

Keywords: (experiences OR attitude* OR awareness OR perspective* OR belief* OR decision* OR preference* OR view*) OR Subject heading terms: (attitude/ OR exp attitude to health/ OR exp attitude to pregnancy/ OR exp patient attitude OR exp patient decision making)

AND

Keywords: (Down* syndrome adj3 screen* OR trisom* adj3 screen* OR aneuploid* adj3 screen*) OR (NIPT or microarray or array CGH or fetal exome or foetal exome or prenatal exome) OR ((prenatal or fetal or foetal) adj3 genom*) OR ((fetal or foetal) adj4 screen*) OR (pregnan* adj4 sickle cell) OR Subject heading terms: (sickle cell anemia/ OR down syndrome/ OR exp chromosome disorder/ OR exp noninvasive prenatal testing/ OR exp prenatal screening/)
