# Supplementary material for: Are People From Black Communities Proportionately Represented in UK and US Studies Examining Views on Screening and Diagnostic Genetic Testing in Pregnancy? A Scoping Review
Source: BJOG. 2025 Apr 29;132(13):1956–65. doi: 10.1111/1471-0528.18195 (PMC12592762; doi:10.1111/1471-0528.18195)
Supplement: Supplementary file 2 — Table S1. Study characteristics of all studies included in the final review. [file BJO-132-1956-s001.docx]

**Table S1**. Study characteristics of all studies included in the final review.

| **Reference, country** | **Study aim** | **Prenatal test, population sampled** | **Design and data collection** | **Racial/ ethnic groups included** | **Black participants included (Yes/No), % of study sample that is Black** | **% of Black participants meets national population* (Yes/No)** |
| --- | --- | --- | --- | --- | --- | --- |
| Agatisa et al. (2015)^1^, US | To explore opinions about the use of NIPT to assess the risk of sex chromosome aneuploidies and microdeletion syndromes | NIPT for sex chromosome aneuploidies amongst pregnant women and women who had recently had a baby | Qualitative design, focus groups | Black or African American, White, Other/multi-ethnic | Yes, 23% | Yes |
| Agatisa et al. (2018)^2^, US | To understand perceptions of decision-making around expanded applications of cffDNA screening | NIPT amongst pregnant women | Qualitative design, focus groups | Asian, Black, White-non-Hispanic, White-Hispanic, Other/multiracial | Yes, 18% | Yes |
| Ahmed et al. (2014)^3^, UK | To explore women's understandings of how health professionals should facilitate informed screening choices | Prenatal screening amongst pregnant women | Qualitative design, semi-structured interviews | African, British White, Caribbean, Chinese and Pakistani | Yes, 30% | Yes |
| Allyse et al. (2014)^4^, US | To determine views on NIPT in order to help estimate uptake. | NIPT amongst adults | Quantitative design, surveys | African American or Black, American Indian or Alaska Native, Asian, Native Hawaiian/ other Pacific Islander, White | Yes, 8% | No |
| Allyse et al. (2015)^5^, US | To understand opinions on prenatal testing technologies including NIPT | NIPT amongst adults | Quantitative design, surveys | American Indian or Alaska Native, Asian, Black or African American, Native Hawaiian or Other Pacific Islander, White | Yes, 8% | No |
| Atkin et al. (2015)^6^, UK | To understand fathers' experiences and expectations of sickle cell antenatal screening | Prenatal screening for sickle cell amongst fathers | Qualitative design, semi-structured interviews | Asian, Black, Mixed | Yes, 88% | Yes |
| Boardman et al. (2019)^7^, UK | To explore views and reproductive decisions of people at risk of transmitting thalassaemia | Prenatal screening for thalassaemia amongst carriers of, those affected by and those with thalassaemia in the family | Qualitative design, semi-structured interviews | Asian, European | No, 0% | No |
| Bryant et al. (2015)^8^, US | To investigate understanding of prenatal testing options and experiences of testing | Prenatal testing amongst pregnant women | Quantitative design, surveys | African American/Black, Caucasian/White, Latina (English-speaking), Latina (Spanish-speaking), Other | Yes, 18% | Yes |
| Calonico et al. (2016)^9^, US | To assess preferences regarding prenatal testing for microdeletion and microduplication conditions | Prenatal testing amongst pregnant women | Quantitative design, surveys | African American, Asian, Asian Indian, Caucasian, Hispanic, Other | Yes, 3% | No |
| Chen et al. (2015)^10^, US | To explore interests in prenatal genetic testing for autism and reproductive decisions among parents of children with autism | Prenatal testing amongst parents with at least one child with ASD | Qualitative design, semi-structured interviews | African American, Asian, Hispanic | Yes, 7% | No |
| Cheung et al. (2019)^11^, US | To identify attitudes and perception of genetic services in the Somali community of Minnesota | Prenatal testing amongst healthcare providers and community members | Qualitative design, semi-structured interviews | Somali, White | Yes, 73% | Yes |
| Cote-Arsenault et al. (2016)^12^, US | To describe parents' lived experience of continuing pregnancy with a lethal fetal diagnosis | Prenatal diagnosis of lethal conditions amongst parents | Qualitative design, semi-structured interviews | Asian/Pacific Islander, African American, Caucasian, Hispanic | Yes, 17% | Yes |
| Dheensa et al. (2014)^13^, UK | To explore what men who attend antenatal appointments want from screening and from midwives | Prenatal screening amongst fathers | Qualitative design, semi-structured interviews | White British | No, 0% | No |
| Farrell et al. (2014)^14^, US | To explore views towards current and future applications of NIPT | NIPT amongst pregnant women | Quantitative design, surveys | African American, Asian, Hispanic, White, Other | Yes, 16% | Yes |
| Farrell et al. (2014)^15^, US | To explore perspectives about NIPT and factors considered during decision making about its use | NIPT amongst women who had received prenatal care | Qualitative design, focus groups | American Indian/Alaskan Native, Asian, Black or African American, Hispanic, Native Hawaiian/Other Pacific Islander, White, Other/Multiethnic | Yes, 32% | Yes |
| Farrell et al. (2016)^16^, US | To identify factors that affect women’s decision-making about NIPT | NIPT amongst women who had had NIPT | Quantitative design, surveys | American Indian/Alaskan Native Asian, Black or African American, White, Other Hispanic | Yes, 4% | No |
| Farrell et al. (2019)^17^, US | To explore partners’ perspectives about the use of cfDNA screening. | NIPT amongst partners | Qualitative design, semi-structured interviews | Asian, Black or African American, White | Yes, 4% | No |
| Farrell et al. (2020)^18^, US | To identify decision‐making needs and preferences with respect to cfDNA | NIPT amongst pregnant women | Qualitative design, semi-structured interviews | Hispanic, Black, non‐Hispanic White | Yes, 4% | No |
| Farrell et al. (2020)^19^, US | To explore patients' decision-making regarding prenatal genetic screening and diagnostic testing. | Prenatal testing amongst pregnant women | Qualitative design, semi-structured interviews | Asian, Black, White, Other | Yes, 7% | No |
| Farrell et al. (2021)^20^, US | To evaluate knowledge and decision-making preferences for expanded carrier and aneuploidy prenatal screening. | Prenatal screening for expanded carrier and aneuploidy screening amongst pregnant women | Quantitative design, surveys | Asian, American Indian or Alaska Native, Black, Hispanic or Latino, White, Other | Yes, 6% | No |
| Floyd et al. (2016)^21^, US | To address the relative lack of knowledge about diverse women’s views of cfDNA screening | NIPT amongst pregnant women | Qualitative design, semi-structured interviews | Hispanic/Latina, Non-Hispanic Asian, Non-Hispanic White | No, 0% | No |
| Gammon et al. (2020)^22^, US | To explore experiences of receiving high-risk cfDNA screening results | NIPT amongst pregnant women | Qualitative design, semi-structured interviews | Asian, Latina, White | No, 0% | No |
| Griffin et al. (2023)^23^, US | To understand decision-making amongst women offered NIPS | NIPT amongst pregnant women | Quantitative design, surveys | African American, Asian, Hispanic or Latino, White, Other | Yes, 6% | No |
| Grinshpun-Cohen et al. (2015)^24^, US | To examine the factors that predict amniocentesis uptake among pregnant women of advanced maternal age | Amniocentesis amongst pregnant women | Quantitative design, surveys | Caucasian, African American, Asian, Hispanic | Yes, 6% | No |
| Hammond et al. (2020)^25^, UK | To understand management of uncertainty after identification of fetal structural anomaly | Prenatal screening for structural anomalies amongst parents | Qualitative design, semi-structured interviews | Asian/Asian British, Black/Black British, Caucasian, Other | No, 0% | No |
| Hammond et al. (2022)^26^, UK | To develop a set of attributes for a discrete-choice experiment to examine parents’ preferences for tests that may reveal uncertain test results | Exome sequencing amongst parents and healthcare professionals | Mixed methods design, semi-structured interviews and surveys | Asian, Black, White, Other | No, 0% | No |
| Harris et al. (2020)^27^, US | To understand parents’ prenatal experience and identify strategies to improve support | Prenatal testing amongst pregnant women and partners | Qualitative design, semi-structured interviews | Black or African American, Hispanic or Latino, White (European), White (Middle Eastern), | Yes, 11% | No |
| Higuchi et al. (2016)^28^, US | To assess the impact of NIPT on support for prenatal testing, decision-making about testing, and beliefs about DS | NIPT for DS amongst pregnant women | Quantitative design, surveys | Caucasian/White, Asian, African American/Black, Other | Yes, 5% | No |
| Hill et al. (2014)^29^, UK | Explore views and attitudes to NIPD of people at risk of having a child with sickle cell, thalassaemia and CF | NIPD for sickle cell, thalassaemia and CF amongst charity members, people seen in a genetics clinic, and pregnant women | Mixed methods design, interviews and focus groups | Asian, Black, Mixed, White, Other | Yes, 25% | Yes |
| Hill et al. (2017)^30^, UK | To determine preferences for key attributes of prenatal diagnostic tests for sickle cell and examine views on NIPD | Diagnostic testing for sickle cell amongst carriers of/ people affected by sickle cell | Quantitative design, surveys | African/Caribbean, Other | Yes, 93% | Yes |
| Igel et al. (2020)^31^, US | To investigate decision-making around diagnostic testing for pregnant women referred for genetic counseling. | Prenatal diagnostic testing amongst pregnant women | Quantitative design, surveys | African‐American, Asian, Caucasian, Hispanic, Mixed | Yes, 23% | Yes |
| Kalynchuk et al. (2015)^32^, US | To examine the opinions of expectant parents regarding prenatal whole-exome sequencing | Exome sequencing amongst pregnant women and partners | Quantitative design, surveys | Asian, Black, White, Other | Yes, 15% | Yes |
| Kellogg et al. (2014)^33^, US | To assess how mothers of children with DS perceive NIPT | NIPT for DS amongst mothers | Quantitative design, surveys | Black, Asian, White, Hispanic | No, 0% | No |
| Kelly-Hedrick et al. (2023)^34^, US | To explore views on receiving abnormal results from prenatal screening | Prenatal screening amongst pregnant women | Quantitative design, surveys | Black, White, Other | Yes, 40% | Yes |
| Kernie et al. (2022)^35^, US | To evaluate the experiences of individuals who chose to receive prenatal genomic sequencing (pGS) for fetuses with congenital structural anomalies | Prenatal genomic sequencing for individuals who received research results of prenatal sequencing | Qualitative design, semi-structured interviews | Asian, Black, More than one race, White | Yes, 3% | No |
| Lewis et al. (2014)^36^, UK | To assess views and likely uptake of NIPT for trisomy 21 among potential service users in the UK. | NIPT for DS amongst pregnant women and members of online parent forums | Quantitative design, surveys | Asian/Asian British, Black/Black British, Mixed, White/White British, Other | Yes, 2% | No |
| Lewis et al. (2014a)^37^, UK | To explore experiences of using NIPD for single gene disorders. | NIPD for single gene disorders amongst women offered NIPD | Qualitative design, semi-structured interviews | Mixed, White | No, 0% | No |
| Lewis et al. (2016)^38^, UK | To explore attitudes towards NIPT and determine factors influencing its uptake | NIPT for aneuploidy amongst women offered NIPT | Qualitative design, semi-structured interviews | Asian/Asian British, Black/Black British, White/White British, Mixed, Other | Yes, 7% | Yes |
| Lewis et al. (2016a)^39^, UK | To assess women’s experience of being offered NIPT | NIPT for DS amongst women with a DS screening risk > 1:1000 | Quantitative design, surveys | Asian/Asian British, Black/Black British, White/White British, Mixed | Yes, 7% | Yes |
| Lewis et al. (2016b)^40^, UK | To validate a modified instrument for assessing informed choice among women offered NIPT | NIPT for DS amongst women who had accepted DS screening | Mixed methods design, semi-structured interviews and surveys | Asian/Asian British, Black/Black British, White/White British, Mixed, Other | Yes, 7% | Yes |
| Lewis et al. (2017)^41^, UK | To assess rates of informed choice among women offered NIPT for aneuploidy | NIPT for aneuploidy amongst pregnant women with a DS screening risk > 1:150 | Quantitative design, surveys | Asian/Asian British, Black/Black British, White/White British, Mixed, Other | Yes, 9% | Yes |
| McInnes-Dean et al. (2024)^42^, UK | To explore views and experiences of prenatal exome sequencing | Prenatal exome sequencing (pES) amongst parents offered pES | Qualitative design, semi-structured interviews | Asian, Black, Mixed, White, Other | Yes, 4% | No |
| Mellis et al. (2022)^43^, UK | To explore parents' and professionals' views and experiences of prenatal exome sequencing | Exome sequencing amongst parents and healthcare professionals | Qualitative design, semi-structured interviews | Asian, White | No, 0% | No |
| Meredith et al. (2024)^44^, US | To understand impact of clinician bias on parental prenatal screening experiences | Prenatal testing for DS amongst parents | Mixed methods design, surveys with qualitative and quantitative components | American Indian or Alaska Native, Asian, Black, Native Hawaiian or other Pacific Islander, White, Unspecified | Yes, 2% | No |
| Molina et al. (2019)^45^, US | To investigate preferred approach to prenatal genetic testing decision making | Prenatal testing amongst pregnant women | Quantitative design, surveys | Asian, Black, Latina (English speaking), Latina (Spanish speaking), White, Other | Yes, 16% | Yes |
| Norton et al. (2014)^46^, US | To assess attitudes towards prenatal screening and diagnostic testing for congenital disorders | Prenatal testing for DS, fragile X, CF, spinal muscular atrophy, phenylketonuria and CHD amongst women who had delivered healthy infants | Qualitative design, semi-structured interviews | African American, Asian, Caucasian, Latina, | Yes, 7% | No |
| Nuccio et al. (2015)^47^, US | To determine perception of miscarriage risk associated with amniocentesis before and after genetic counseling | Amniocentesis amongst pregnant women | Quantitative design, surveys | African American, Asian, Caucasian, Hispanic, | Yes, 24% | Yes |
| Outram et al. (2022)^48^, US | To understand parental expectations of diagnostic prenatal genomic sequencing and the value of the results to families. | Prenatal genomic sequencing amongst parents who had had prenatal genomic sequencing | Qualitative design, semi-structured interviews | Asian, Asian/White, Hispanic, White | No, 0% | No |
| Pham et al. (2023)^49^, US | To determine attitudes towards prenatal diagnosis and preferences on types of prenatal therapies for haemophilia. | Prenatal diagnosis of haemophilia amongst members of the haemophilia community | Quantitative design, surveys | Asian, Black/African, White | Yes, 1% | No |
| Piechan et al. (2016)^50^, US | To evaluate patients’ basic understanding of NIPT and satisfaction with the testing process | NIPT amongst pregnant women who had had NIPT | Quantitative design, surveys | African American or Black, Asian/Pacific Islander, Hispanic or Latina, White, Other | Yes, 8% | No |
| Prince et al. (2021)^51^, US | To understand views towards genetic screening and testing, specifically for sickle cell disease | Prenatal testing for sickle cell amongst pregnant women | Quantitative design, surveys | American Indian or Alaska Native, Asian, Native Hawaiian or other Pacific Islander, Black, Hispanic or Latino, White, Other | Yes, 41% | Yes |
| Quinlan-Jones et al. (2017)^52^, UK | To explore parental experiences of whole exome sequencing (WES) for prenatal diagnosis | Whole exome sequencing for women who had received a fetal anomaly diagnosis on ultrasound | Qualitative design, semi-structured interviews | Asian, Black African, Caucasian | Yes, 8% | Yes |
| Reese et al. (2018)^53^, US | To explore attitudes toward and uptake of current prenatal genetic screening and diagnostic testing for fetal aneuploidy in twin pregnancies | Prenatal testing for fetal aneuploidy amongst pregnant women | Mixed methods design, semi-structured interviews and surveys | African American, Asian, Caucasian, Hispanic, Other | Yes, 21% | Yes |
| Richards et al. (2015)^54^, US | To compare genetic versus ultrasound modalities to see how pregnant patients perceive risk following uncertain results. | Prenatal screening amongst pregnant women | Quantitative design, surveys | African‐American, Asian, Caucasian, Hispanic, Other | Yes, 22% | Yes |
| Riches et al. (2023)^55^, US | To explore experiences of prenatal screening education and its delivery | Prenatal screening amongst women offered it in the previous 12 months | Qualitative design, semi-structured interviews | Black or African American, Hispanic, Pacific Islander | Yes, 33% | Yes |
| Riggan et al. (2020)^56^, US | To investigate family experiences and attitudes about receiving the diagnosis of sex chromosome aneuploidy (SCA) | Diagnostic testing for sex chromosome aneuploidies amongst individuals/ parents of individuals with | Quantitative design, surveys | African American, Asian, Hispanic or Latino, White, Other | Yes, 1% | No |
| Riggan et al. (2021)^57^, US | To explore experiences of parents receiving a prenatal diagnosis of a fetus with SCA | Diagnostic testing for sex chromosome aneuploidies amongst parents | Qualitative design, surveys | Asian, Hispanic, Non-Hispanic Black, Non-Hispanic White, Other | Yes, 1% | No |
| Riggan et al. (2024)^58^, US | To explore experiences and perceptions of prenatal cfDNA screening. | NIPT amongst Black and Hispanic patients receiving prenatal care | Qualitative design, semi-structured interviews | Hispanic, Hispanic Black, non‐Hispanic Black, Not Reported/Other | Yes, 54% | Yes |
| Rothwell et al., (2017)^59^, US | To explore the experiences of women who received positive results from expanded carrier screening tests | Prenatal screening | Qualitative design, semi-structured interviews | Hispanic, Non‐Hispanic White, White | No, 0% | No |
| Rubel et al. (2017)^60^, US | To assess how participants receiving abnormal prenatal genetic testing results seek information and understand the implications of results | Microarray | Qualitative design, semi-structured interviews | Asian, Asian/White, Black, White | Yes, 3% | No |
| Sayres et al. (2014)^61^, US | To explore public attitudes towards cffDNA technology and characteristics related to these attitudes. | NIPT for trisomies 13, 18, and 21 amongst members of the public | Quantitative design, surveys | American Indian or Alaska Native, Asian, Black or African-American, Native Hawaiian or Pacific Islander, White | Yes, 8% | No |
| Silcock et al. (2015)^62^, UK | To investigate impact of non-invasive pre-natal testing for DS on informed choice | NIPT for DS amongst healthcare providers and pregnant women | Quantitative design, surveys | Asian/Asian British, Black/Black British,White British/European, Mixed, Other | Yes, 4% | No |
| Skirton et al. (2015)^63^, UK | To explore the views of carriers of recessive conditions about NIPD | NIPT amongst carriers of CF, thalassaemia, SMA and sickle cell | Qualitative design, semi-structured interviews | British Asian, African-American/White, Persian, White British, White other | Yes, 4% | No |
| Sofer et al. (2020)^64, 65^, US | To explore views on fetal sex determination methods, knowledge of cfDNA and differences of sex development (DSD) | NIPT for differences of sex development amongst pregnant women and partners | Quantitative design, surveys | American Indian, Asian, Black/African-American, Native Hawaiian, White/Caucasian, Other | Yes, 9% | No |
| Stevens et al. (2019), US | To explore knowledge and attitudes of prenatal genetic counselling among parents of children with sickle cell | Prenatal testing amongst parents of children with sickle cell | Quantitative design, surveys | Black | Yes, 93% | Yes |
| Sullivan at al. (2019)^66^, US | To assess pregnant women’s views and preferences on non-invasive prenatal whole genome sequencing. | NIPD amongst pregnant women | Quantitative design, surveys | American Indian, Asian, Black, Hispanic or Latina, Middle Eastern, Multi-racial, Native Hawaiian, White | Yes, 11% | No |
| Talati et al. (2021)^67^, US | To evaluate associations between prenatal exome sequencing and psychological outcomes among women with an anomalous pregnancy | Exome sequencing amongst parents | Mixed methods design, surveys with qualitative and quantitative components | Asian, Black, Hispanic, Native American/Alaskan, White, Other | Yes, 7% | No |
| Talati et al. (2022)^68^, US | To understand motivations for trio‐exome sequencing (ES) for fetal anomalies with a negative standard genetic diagnosis. | Exome sequencing amongst parents with a pregnancy complicated by either isolated or multiple congenital anomalies | Mixed methods design, semi-structured interviews and surveys | African American, Asian/Pacific Islander, Hispanic, Native American, White | Yes, 12% | No |
| Thomas et al. (2024)^69^, US | To explore Black women’s attitudes towards NIPT screening for sickle cell | NIPT for sickle cell amongst pregnant women | Qualitative design, semi-structured interviews | African, African American, Afro-Caribbean, Afro-Latinx, Black | Yes, 100% | Yes |
| Tiller et al. (2015)^70^, US | To determine the impact of NIPT on invasive procedure utilization in a managed care setting | NIPT amongst pregnant women | Quantitative design, surveys | Asian, Black, Hispanic, White, Other | Yes, 3% | No |
| Walser et al. (2015)^71^, US | To understand educational needs regarding prenatal CMA testing | Microarray amongst patients who had had microarray | Quantitative design, surveys | Black/African American, Asian, White, More than one race, Other | Yes, 2% | No |
| Walser et al. (2016)^72^, US | To explore how couples’ understanding of chromosomal microarray analysis (CMA) results impacts decision making | Microarray amongst parents | Qualitative design, semi-structured interviews | Asian, Black, Hispanic/Latino, White | Yes, 3% | No |
| Werner-Lin et al. (2016)^73^, US | To explore experiences of receiving abnormal or uncertain results from prenatal microarray testing | Microarray amongst parents | Qualitative design, semi-structured interviews | Hispanic/Latino, White, Other | No, 0% | No |
| Whitehead et al. (2022)^74^, US | To explore experiences of parents whose children had prenatal findings of differences of sex development (DSD) | Prenatal testing for differences of sex development amongst parents | Mixed methods design, semi-structured interviews and surveys | Asian, White, Other | No, 0% | No |
| Wittman et al. (2016)^75^, US | To determine patient perception of residual risk after receiving a negative NIPT result | NIPT amongst women consented for NIPT | Quantitative design, surveys | Asian, African American, Hispanic, Non-Hispanic white, Other | Yes, 17% | Yes |
| Wou et al. (2018)^76^, US | To investigate the views and experiences of couples who underwent prenatal whole‐exome sequencing (WES) for fetal anomalies | Exome/genome sequencing amongst participants in the Fetal Sequencing Study who had genetic testing for fetal anomalies | Qualitative design, semi-structured interviews | Asian, African American, Caucasian, Caucasian/Hispanic, Hispanic, Western European | Yes, 9% | No |

1. Agatisa PK, Mercer MB, Leek AC, Smith MB, Philipson E, Farrell RM. A first look at women's perspectives on noninvasive prenatal testing to detect sex chromosome aneuploidies and microdeletion syndromes. Prenat Diagn. 2015;35(7):692-8.

2. Agatisa PK, Mercer MB, Mitchum A, Coleridge MB, Farrell RM. Patient-Centered Obstetric Care in the Age of Cell-Free Fetal DNA Prenatal Screening. J Patient Exp. 2018;5(1):26-33.

3. Ahmed S, Bryant LD, Tizro Z, Shickle D. Is advice incompatible with autonomous informed choice? Women's perceptions of advice in the context of antenatal screening: a qualitative study. Health Expect. 2014;17(4):555-64.

4. Allyse M, Sayres LC, Goodspeed TA, Cho MK. Attitudes towards non-invasive prenatal testing for aneuploidy among US adults of reproductive age. J Perinatol. 2014;34(6):429-34.

5. Allyse M, Sayres LC, Goodspeed T, Michie M, Cho MK. "Don't Want No Risk and Don't Want No Problems": Public Understandings of the Risks and Benefits of Non-Invasive Prenatal Testing in the United States. AJOB Empir Bioeth. 2015;6(1):5-20.

6. Atkin K, Berghs M, Dyson S. 'Who's the guy in the room?' Involving fathers in antenatal care screening for sickle cell disorders. Soc Sci Med. 2015;128:212-9.

7. Boardman FK, Clark C, Jungkurth E, Young PJ. Social and cultural influences on genetic screening programme acceptability: A mixed-methods study of the views of adults, carriers, and family members living with thalassemia in the UK. J Genet Couns. 2020;29(6):1026-40.

8. Bryant AS, Norton ME, Nakagawa S, Bishop JT, Pena S, Gregorich SE, et al. Variation in Women's Understanding of Prenatal Testing. Obstet Gynecol. 2015;125(6):1306-12.

9. Calonico E, Blumenfeld YJ, Hudgins L, Taylor J. Patient preferences for prenatal testing of microdeletion and microduplication syndromes. Prenat Diagn. 2016;36(3):244-51.

10. Chen LS, Xu L, Dhar SU, Li M, Talwar D, Jung E. Autism spectrum disorders: a qualitative study of attitudes toward prenatal genetic testing and termination decisions of affected pregnancies. Clin Genet. 2015;88(2):122-8.

11. Cheung FY, Pratt R, Shire A, Bigalke L, Ahmed Z, Zierhut H. Developing culturally informed genetic services for the Somali immigrants in Minnesota. J Genet Couns. 2019;28(4):887-96.

12. Côté-Arsenault D, Denney-Koelsch E. "Have no regrets:" Parents' experiences and developmental tasks in pregnancy with a lethal fetal diagnosis. Soc Sci Med. 2016;154:100-9.

13. Dheensa S, Metcalfe PA, Williams R. What do men want from antenatal screening? Findings from an interview study in England. Midwifery. 2015;31(1):208-14.

14. Farrell RM, Agatisa PK, Nutter B. What women want: lead considerations for current and future applications of noninvasive prenatal testing in prenatal care. Birth. 2014;41(3):276-82.

15. Farrell RM, Mercer MB, Agatisa PK, Smith MB, Philipson E. It's More Than a Blood Test: Patients' Perspectives on Noninvasive Prenatal Testing. J Clin Med. 2014;3(2):614-31.

16. Farrell RM, Agatisa PK, Mercer MB, Mitchum A, Coleridge M. Expanded indications for noninvasive prenatal genetic testing: Implications for the individual and the public. Ethics, Medicine and Public Health. 2016;2(3):383-91.

17. Farrell RM, Mercer M, Agatisa PK, Coleridge MB. Balancing Needs and Autonomy: The Involvement of Pregnant Women's Partners in Decisions About cfDNA. Qual Health Res. 2019;29(2):211-21.

18. Farrell RM, Agatisa PK, Michie MM, Greene A, Ford PJ. The personal utility of cfDNA screening: Pregnant patients' experiences with cfDNA screening and views on expanded cfDNA panels. J Genet Couns. 2020;29(1):88-96.

19. Farrell RM, Pierce M, Collart C, Edmonds BT, Chien E, Coleridge M, et al. Making the most of the first prenatal visit: The challenge of expanding prenatal genetic testing options and limited clinical encounter time. Prenat Diagn. 2020;40(10):1265-71.

20. Farrell RM, Pierce M, Collart C, Yao M, Coleridge M, Chien EK, et al. Decision-making for prenatal genetic screening: how will pregnant women navigate a growing number of aneuploidy and carrier screening options? BMC Pregnancy Childbirth. 2021;21(1):806.

21. Floyd E, Allyse MA, Michie M. Spanish- and English-Speaking Pregnant Women's Views on cfDNA and Other Prenatal Screening: Practical and Ethical Reflections. J Genet Couns. 2016;25(5):965-77.

22. Gammon BL, Jaramillo C, Riggan KA, Allyse M. Decisional regret in women receiving high risk or inconclusive prenatal cell-free DNA screening results. J Matern Fetal Neonatal Med. 2020;33(8):1412-8.

23. Griffin E, Hooker G, Grace M, Kaphingst K, Velez Edwards D, Zhao Z, et al. What knowledge is required for an informed choice related to non-invasive prenatal screening? J Genet Couns. 2023;32(4):812-22.

24. Grinshpun-Cohen J, Miron-Shatz T, Rhee-Morris L, Briscoe B, Pras E, Towner D. A Priori Attitudes Predict Amniocentesis Uptake in Women of Advanced Maternal Age: A Pilot Study. J Health Commun. 2015;20(9):1107-13.

25. Hammond J, Klapwijk JE, Hill M, Lou S, Ormond KE, Diderich KEM, et al. Parental experiences of uncertainty following an abnormal fetal anomaly scan: Insights using Han's taxonomy of uncertainty. J Genet Couns. 2021;30(1):198-210.

26. Hammond J, Klapwijk JE, Riedijk S, Lou S, Ormond KE, Vogel I, et al. Assessing women's preferences towards tests that may reveal uncertain results from prenatal genomic testing: Development of attributes for a discrete choice experiment, using a mixed-methods design. PLoS One. 2022;17(1):e0261898.

27. Harris KW, Brelsford KM, Kavanaugh-McHugh A, Clayton EW. Uncertainty of Prenatally Diagnosed Congenital Heart Disease: A Qualitative Study. JAMA Netw Open. 2020;3(5):e204082.

28. Higuchi EC, Sheldon JP, Zikmund-Fisher BJ, Yashar BM. Non-invasive prenatal screening for trisomy 21: Consumers' perspectives. Am J Med Genet A. 2016;170a(2):375-85.

29. Hill M, Compton C, Karunaratna M, Lewis C, Chitty L. Client views and attitudes to non-invasive prenatal diagnosis for sickle cell disease, thalassaemia and cystic fibrosis. J Genet Couns. 2014;23(6):1012-21.

30. Hill M, Oteng-Ntim E, Forya F, Petrou M, Morris S, Chitty LS. Preferences for prenatal diagnosis of sickle-cell disorder: A discrete choice experiment comparing potential service users and health-care providers. Health Expect. 2017;20(6):1289-95.

31. Igel CM, Rabin-Havt S, Estrada Trejo F, Doulaveris G, Eisenberg R, Fazzari M, et al. Patient attitudes toward prenatal diagnostic testing during antenatal care in an urban population. Prenat Diagn. 2021;41(7):888-95.

32. Kalynchuk EJ, Althouse A, Parker LS, Saller DN, Jr., Rajkovic A. Prenatal whole-exome sequencing: parental attitudes. Prenat Diagn. 2015;35(10):1030-6.

33. Kellogg G, Slattery L, Hudgins L, Ormond K. Attitudes of mothers of children with down syndrome towards noninvasive prenatal testing. J Genet Couns. 2014;23(5):805-13.

34. Kelly-Hedrick M, Geller G, Jelin AC, Gross MS. Perceived Value of Prenatal Ultrasound Screening: A Survey of Pregnant Women. Matern Child Health J. 2023;27(1):101-10.

35. Kernie CG, Wynn J, Rosenbaum A, de Voest J, Galloway S, Giordano J, et al. Information is power: The experiences, attitudes and needs of individuals who chose to have prenatal genomic sequencing for fetal anomalies. Prenat Diagn. 2022;42(7):947-54.

36. Lewis C, Hill M, Silcock C, Daley R, Chitty LS. Non-invasive prenatal testing for trisomy 21: a cross-sectional survey of service users' views and likely uptake. Bjog. 2014;121(5):582-94.

37. Lewis C, Hill M, Chitty LS. Non-invasive prenatal diagnosis for single gene disorders: experience of patients. Clin Genet. 2014;85(4):336-42.

38. Lewis C, Hill M, Chitty LS. A qualitative study looking at informed choice in the context of non-invasive prenatal testing for aneuploidy. Prenat Diagn. 2016;36(9):875-81.

39. Lewis C, Hill M, Chitty LS. Women's Experiences and Preferences for Service Delivery of Non-Invasive Prenatal Testing for Aneuploidy in a Public Health Setting: A Mixed Methods Study. PLoS One. 2016;11(4):e0153147.

40. Lewis C, Hill M, Skirton H, Chitty LS. Development and validation of a measure of informed choice for women undergoing non-invasive prenatal testing for aneuploidy. Eur J Hum Genet. 2016;24(6):809-16.

41. Lewis C, Hill M, Chitty LS. Offering non-invasive prenatal testing as part of routine clinical service. Can high levels of informed choice be maintained? Prenat Diagn. 2017;37(11):1130-7.

42. McInnes-Dean H, Mellis R, Daniel M, Walton H, Baple EL, Bertoli M, et al. 'Something that helped the whole picture': Experiences of parents offered rapid prenatal exome sequencing in routine clinical care in the English National Health Service. Prenat Diagn. 2024;44(4):465-79.

43. Mellis R, Tapon D, Shannon N, Dempsey E, Pandya P, Chitty LS, et al. Implementing a rapid fetal exome sequencing service: What do parents and health professionals think? Prenat Diagn. 2022;42(6):783-95.

44. Meredith S, Weiss S, Kleinert HL, Tyrrell CA. The impact of implicit and explicit bias about disabilities on parent experiences and information provided during prenatal screening and testing. Disabil Health J. 2024;17(1):101514.

45. Molina F, Dehlendorf C, Gregorich SE, Kuppermann M. Women's preferences for and experiences with prenatal genetic testing decision making: Sociodemographic disparities in preference-concordant decision making. Patient Educ Couns. 2019;102(3):595-601.

46. Norton ME, Nakagawa S, Kuppermann M. Women’s Attitudes Regarding Prenatal Testing for a Range of Congenital Disorders of Varying Severity. Journal of Clinical Medicine [Internet]. 2014; 3(1):[144-52 pp.].

47. Nuccio R, Hashmi SS, Mastrobattista J, Noblin SJ, Refuerzo J, Smith JL, et al. Influence of anchoring on miscarriage risk perception associated with amniocentesis. J Genet Couns. 2015;24(2):278-84.

48. Outram SM, Brown JEH, Zamora AN, Sahin-Hodoglugil N, Ackerman SL. Parental Hopes and Understandings of the Value of Prenatal Diagnostic Genomic Sequencing: A Qualitative Analysis. Front Genet. 2022;13:883225.

49. Pham QDM, Thomson SM, Schaible BN, Mills KD, Atala A, Porada CD, et al. Acceptability of prenatal diagnosis and prenatal treatment of haemophilia using cell and gene therapies within US haemophilia community. Haemophilia. 2023;29(4):1024-31.

50. Piechan JL, Hines KA, Koller DL, Stone K, Quaid K, Torres-Martinez W, et al. NIPT and Informed Consent: an Assessment of Patient Understanding of a Negative NIPT Result. J Genet Couns. 2016;25(5):1127-37.

51. Prince A, Cruz-Bendezú A, Gunawansa N, Wade J, Coleman-Cowger VH, Schulkin J, et al. Practices of sickle cell disease genetic screening and testing in the prenatal population. J Neonatal Perinatal Med. 2021;15(4):745-51.

52. Quinlan-Jones E, Hillman SC, Kilby MD, Greenfield SM. Parental experiences of prenatal whole exome sequencing (WES) in cases of ultrasound diagnosed fetal structural anomaly. Prenat Diagn. 2017;37(12):1225-31.

53. Reese KM, Czerwinski J, Darilek S, Johnson A, Jones M, Singletary CN. Attitudes Toward and Uptake of Prenatal Genetic Screening and Testing in Twin Pregnancies. J Genet Couns. 2018;27(5):1238-47.

54. Richards EG, Sangi-Haghpeykar H, McGuire AL, Van den Veyver IB, Fruhman G. Pregnant patients' risk perception of prenatal test results with uncertain fetal clinical significance: ultrasound versus advanced genetic testing. Prenat Diagn. 2015;35(12):1213-7.

55. Riches NO, Johnson EP, Subramaniam A, Vora NL, Hardisty E, LaRiviere K, et al. Understanding the experiences and perspectives of prenatal screening among a diverse cohort. Prenat Diagn. 2023;43(5):605-12.

56. Riggan KA, Close S, Allyse MA. Family experiences and attitudes about receiving the diagnosis of sex chromosome aneuploidy in a child. Am J Med Genet C Semin Med Genet. 2020;184(2):404-13.

57. Riggan KA, Gross B, Close S, Weinberg A, Allyse MA. Prenatal Genetic Diagnosis of a Sex Chromosome Aneuploidy: Parent Experiences. J Genet Couns. 2021;30(5):1407-17.

58. Riggan KA, Barwise A, Yap JQ, Condon N, Allyse MA. Patient experiences with prenatal cell-free DNA screening in a safety net setting. Prenat Diagn. 2024;44(4):409-17.

59. Rothwell E, Johnson E, Mathiesen A, Golden K, Metcalf A, Rose NC, et al. Experiences among Women with Positive Prenatal Expanded Carrier Screening Results. J Genet Couns. 2017;26(4):690-6.

60. Rubel MA, Werner-Lin A, Barg FK, Bernhardt BA. Expert Knowledge Influences Decision-Making for Couples Receiving Positive Prenatal Chromosomal Microarray Testing Results. Cult Med Psychiatry. 2017;41(3):382-406.

61. Sayres LC, Allyse M, Goodspeed TA, Cho MK. Demographic and experiential correlates of public attitudes towards cell-free fetal DNA screening. J Genet Couns. 2014;23(6):957-67.

62. Silcock C, Liao LM, Hill M, Chitty LS. Will the introduction of non-invasive prenatal testing for Down's syndrome undermine informed choice? Health Expect. 2015;18(5):1658-71.

63. Skirton H, Goldsmith L, Chitty LS. An easy test but a hard decision: ethical issues concerning non-invasive prenatal testing for autosomal recessive disorders. Eur J Hum Genet. 2015;23(8):1004-9.

64. Sofer L, D'Oro A, Rosoklija I, Leeth EA, Goetsch AL, Moses S, et al. Impact of cell-free DNA screening on parental knowledge of fetal sex and disorders of sex development. Prenat Diagn. 2020;40(11):1489-96.

65. Stevens EM, Patterson CA, Tchume-Johnson T, Antiel RM, Flake A, Smith-Whitley K, et al. Parental Attitudes Towards Prenatal Genetic Testing For Sickle Cell Disease. J Pediatr Hematol Oncol. 2019;41(8):579-85.

66. Sullivan HK, Bayefsky M, Wakim PG, Huddleston K, Biesecker BB, Hull SC, et al. Noninvasive Prenatal Whole Genome Sequencing: Pregnant Women's Views and Preferences. Obstet Gynecol. 2019;133(3):525-32.

67. Talati AN, Gilmore KL, Hardisty EE, Lyerly AD, Rini C, Vora NL. Impact of prenatal exome sequencing for fetal genetic diagnosis on maternal psychological outcomes and decisional conflict in a prospective cohort. Genet Med. 2021;23(4):713-9.

68. Talati AN, Gilmore KL, Hardisty EE, Lyerly AD, Rini C, Vora NL. Parental motivations for and adaptation to trio-exome sequencing in a prospective prenatal testing cohort: Beyond the diagnosis. Prenat Diagn. 2022;42(6):775-82.

69. Thomas SP, Fletcher FE, Willard R, Ranson TM, Bonham VL. Patient Perceptions on the Advancement of Noninvasive Prenatal Testing for Sickle Cell Disease among Black Women in the United States. AJOB Empir Bioeth. 2024;15(2):154-63.

70. Tiller GE, Kershberg HB, Goff J, Coffeen C, Liao W, Sehnert AJ. Women's views and the impact of noninvasive prenatal testing on procedures in a managed care setting. Prenat Diagn. 2015;35(5):428-33.

71. Walser SA, Kellom KS, Palmer SC, Bernhardt BA. Comparing genetic counselor's and patient's perceptions of needs in prenatal chromosomal microarray testing. Prenat Diagn. 2015;35(9):870-8.

72. Walser SA, Werner-Lin A, Russell A, Wapner RJ, Bernhardt BA. "Something Extra on Chromosome 5": Parents' Understanding of Positive Prenatal Chromosomal Microarray Analysis (CMA) Results. J Genet Couns. 2016;25(5):1116-26.

73. Werner-Lin A, Barg FK, Kellom KS, Stumm KJ, Pilchman L, Tomlinson AN, et al. Couple's Narratives of Communion and Isolation Following Abnormal Prenatal Microarray Testing Results. Qual Health Res. 2016;26(14):1975-87.

74. Whitehead J, Hirsch J, Rosoklija I, Goetsch Weisman A, Dungan J, Finlayson C, et al. Prenatal detection and evaluation of differences of sex development: A qualitative interview study of parental perspectives and unmet needs. Prenat Diagn. 2022;42(10):1332-42.

75. Wittman AT, Hashmi SS, Mendez-Figueroa H, Nassef S, Stevens B, Singletary CN. Patient Perception of Negative Noninvasive Prenatal Testing Results. AJP Rep. 2016;6(4):e391-e406.

76. Wou K, Weitz T, McCormack C, Wynn J, Spiegel E, Giordano J, et al. Parental perceptions of prenatal whole exome sequencing (PPPWES) study. Prenat Diagn. 2018;38(11):801-11.
