# Supplementary material for: Are People From Black Communities Proportionately Represented in UK and US Studies Examining Views on Screening and Diagnostic Genetic Testing in Pregnancy? A Scoping Review
Source: BJOG. 2025 Apr 29;132(13):1956–65. doi: 10.1111/1471-0528.18195 (PMC12592762; doi:10.1111/1471-0528.18195)
Supplement: Supplementary file 3 — Table S2. Percentage of Black participants included across studies. [file BJO-132-1956-s002.docx]

| **Table S2.** Percentage of Black participants included across studies. | | | |
| --- | --- | --- | --- |
|  |  |  |  |
|  | **All studies** | **US studies** | **UK studies** |
|  |  |  |  |
|  | n = 76 | n = 57 | n = 19 |
| Range | 0-100 | 0-100 | 0-93 |
| Q1 | 2.75 | 3 | 0 |
| Median | 7 | 8 | 4 |
| Q3 | 17.25 | 18 | 8.5 |
| Mean | 15.17 | 15.18 | 15.16 |
| 95% CI | (10.0-20.4) | (9.6-20.8) | (1.8-28.6) |
| *SD* | 22.75 | 21.10 | 27.79 |
|  |  |  |  |
| *Key*: CI = confidence interval; *SD* = standard deviation; Q1 = first quartile; Q3 = third quartile | | | |
